# Vaccines and more: The response of Dark Web marketplaces to the ongoing COVID-19 pandemic

**DOI:** 10.1371/journal.pone.0275288

**Published:** 2022-11-10

**Authors:** Alberto Bracci, Matthieu Nadini, Maxwell Aliapoulios, Damon McCoy, Ian Gray, Alexander Teytelboym, Angela Gallo, Andrea Baronchelli

**Affiliations:** 1 Department of Mathematics, City, University of London, London, United Kingdom; 2 The Alan Turing Institute, British Library, London, United Kingdom; 3 Center for Cybersecurity (CCS), New York Univ, Tandon School of Engineering, Brooklyn, NY, United States of America; 4 Global Intelligence Team, Flashpoint, New York, NY, United States of America; 5 Institute for New Economic Thinking, Oxford Martin School, University of Oxford, Oxford, United Kingdom; 6 Department of Economics, University of Oxford, Oxford, United Kingdom; 7 Department of Finance, Bayes Business School, London, United Kingdom; 8 UCL Centre for Blockchain Technologies, University College London, London, United Kingdom; University of Exeter, UNITED KINGDOM

## Abstract

Early analyses revealed that dark web marketplaces (DWMs) started offering COVID-19 related products (e.g., masks and COVID-19 tests) as soon as the COVID-19 pandemic started, when these goods were in shortage in the traditional economy. Here, we broaden the scope and depth of previous investigations by considering how DWMs responded to an ongoing pandemic after the initial shock. Our dataset contains listings from 194 DWMs collected until July 2021. We start by focusing on vaccines. We find 248 listings offering approved vaccines, like Pfizer/BioNTech and AstraZeneca, as well as vendors offering fabricated proofs of vaccination and COVID-19 passports. Then, we consider COVID-19 related products. We show that, as the regular economy has become able to satisfy the demand of these goods, DWMs have decreased their offer. Next, we analyse the profile of vendors of COVID-19 related products and vaccines. We find that most of them are specialized in a single type of listings and are willing to ship worldwide. Finally, we consider a broader set of listings mentioning COVID-19, in order to assess the general impact of the pandemic on the broader activity of DWMs. Among 10,330 such listings, we show that recreational drugs are the most affected among traditional DWMs product, with COVID-19 mentions steadily increasing since March 2020. We anticipate that our results will be of interest to researchers, practitioners, and law enforcement agencies focused on the study and safeguard of public health.

## Introduction

COVID-19 has caused a worldwide economic and public health crisis, that demanded and stimulated a global response. Hundreds of possible COVID-19 vaccines have been proposed [1] since the first officially approved vaccines in late 2020, like Sputnik [2] and Pfizer/BioNTech [3–5]. The subsequent initial scarcity and unequal distribution of COVID-19 vaccines [6] have generated concerns about illicit trade early on. Interpol warned about illicit offering of COVID-19 vaccines already on December 2, 2020 [7], while Europol confirmed the sale of fake COVID-19 vaccines on dark web marketplaces (DWMs) on December 4, 2020 [8], warning that it “may pose a significant risk to public health”. Understanding how DWMs reacted to the demand for vaccines is therefore crucial to allow policy and public health agencies to be prepared and effectively counteract these threats in the future.

Interpol and Europol’s concerns were validated by early research showing that DWMs have been an important channel to access online illicit trade during the pandemic, with masks, COVID-19 tests, and alleged medicines consistently advertised on these platforms. In a first report [9], 222 COVID-19 related unique listings were registered on April 3*rd*, 2020 in 20 DWMs. In our previous work [10], 788 COVID-19 related listings were observed 9, 464 times between January 1, 2020 and November 16, 2020 in 30 DWMs, showing how DWMs swiftly reacted to shortages and public attention by offering sought-after products like masks and hydroxychloroquine. More recent reports, carried by the Global Initiative and Europol, have suggested that the overall structure of illicit online trading has gained significant benefits from COVID-19 [11, 12].

DWMs are an ideal venue to participate in online illicit activities. They can be easily accessed via specialized browsers, e.g. Tor [13], that hide the identity and location of their users, and offer a variety of illicit goods including drugs, firearms, credit cards, and fake IDs [14]. DWMs drew the attention of hundreds of thousands buyers and sellers over the years, with a trading volume that rapidly reached hundreds of millions United States dollars (USD) per year [15, 16]. The growing popularity of DWMs has attracted the interest of the scientific community, security researchers, and law enforcement agencies. The scientific community has explored the behaviour of DWMs users through comparative analyses [17–23] and case studies [24–26]. Law enforcement agencies have successfully closed several DWMs, seizing millions of USD, and performing dozen of arrests [27–33]. However, DWMs are intrinsically resilient to these interventions [16], also thanks to the emergence of decentralized trade around them [34], and 2020 has been a record year for their revenue [35, 36].

Here, we report on our analysis of 194 DWMs until July 22, 2021. In doing so we extend previous analyses, focused on the immediate reaction of DWMs to the shock caused by the onset of the COVID-19 emergency [10], to consider how DMWs have responded to the ongoing pandemic. Furthermore, the period we cover includes the milestones of COVID-19 vaccines being approved and made available, allowing us to investigate their offer on unregulated markets. We detected a total of 10,330 unique listings that were directly affected by COVID-19, i.e., mentioning COVID-19 either in their body or title. Among these listings, 248 were offering vaccines. It is important to note that a listing does not correspond to the sale of a unit, as sometimes happens for example on Ebay, but corresponds to the availability of multiple units of a product, similarly to what happens for example on Amazon. Listings related to approved vaccines were initially detected on the Invictus marketplace starting from November 17, 2020, almost 2 weeks before their official approval. Also, listings offering a fabricated proof of vaccination were registered on the Hydra marketplace since February 15, 2021. These listings replaced previously identified COVID-19 related products, like PPEs, COVID-19 tests, and guides on how to illicitly obtain COVID-19 relief funds. The availability of these products have decreased with respect to previous observations, with only 187 listings detected between November 2020 and July 2021 against the 788 registered between January and November 2020 [10]. Many vendors selling these products are highly specialised in only a type of product and willing to ship worldwide, thereby increasing the number of potential customers. By analysing all listings mentioning COVID-19, we assess the overall impact of COVID-19 on DWMs. We show that drugs are the only traditional DWMs product to have been indirectly, and increasingly, affected by the pandemic, with vendors mentioning both pandemic related supply issues and delays.

Our results confirm the concerns that several international agencies have expressed regarding the online illicit trade of COVID-19 vaccines, and corroborate the link between the shortage of and public attention on medical products, and their availability on DWMs. In addition, they reveal that DWMs were only partially affected by the pandemic, with mostly drugs related listings explicitly mentioning COVID-19, while other traditional DWMs products, like firearm and fake IDs, were not. To reach a large audience beyond academia, we released a website [37], where we are providing constant updates on the effect of the pandemic on DWMs.

## Data and methods

Our dataset includes the most popular DWMs in 2020 and 2021, such as White House, Empire, Hydra, and DarkMarket [9, 38] and was gathered by Flashpoint [39], a company specializing in online risk intelligence. Note that the landscape of active DWMs is constantly changing: Empire exit scammed, meaning that it closed down without any notice and taking away the deposited funds of its users, on August 23, 2020 [40], while DarkMarket was shut down by Europol on January 12, 2021 [41]. The dataset was obtained by web crawling DWMs, which consists of extracting and downloading data from these websites. To this end, the web crawling pipeline has to overcome strong CAPTCHAs [42] and authenticate into the DWMs of interest. Downloading content from DWMs remains a challenging task, and the objective becomes even harder when the research study requires monitoring multiple DWMs for an extended period of time. Previous research groups have tried establishing a web crawling pipeline through a combination of PHP, curl, and MySQL [43], through the Python library Scrapy [44], and through an automated methodology using the AppleScript language [45]. Despite these efforts, only a few open-source tools are available [42, 46] for crawling DWMs. Therefore researchers, companies, and federal agencies often rely on commercial software, like X-Byte [47], and specialized companies, like Flashpoint [39], to crawl DWMs.

Our DWMs dataset is used to complement and extend the analysis we previously performed for the period between January 2020 and November 2020 [10]. The new dataset also covers the period following the approval of the vaccines and their actual distribution to the population, i.e. Nov 2020 to July 2021, and allows to observe the evolution of COVID-19 related products over the second part of the pandemic. We also add several new DMWs, increasing their number from 30 to 194, and comprehending a total of 10.8 million unique listing titles. Only 84 of these DWMs mentioned COVID-19, 20 DWMs offered COVID-19 related products, and 19 vaccines, see Table 8 in S1 File. Each unique listing is observed at most once per day.

During the considered period of the COVID-19 pandemic, the illicit offer of vaccines constituted one of the biggest threats for global public health. We therefore use a method to detect vaccine listings that ensures the highest possible coverage and accuracy. From the listings, we considered two different text fields: the title and the body (that is, the listing’s detailed description). We then pre-selected all listings which contained, either in the body or title, at least one word from two different lists of keyword. These lists of keywords are shown in Table 1: the first list contains keywords related to vaccines; the second list contains keywords related to COVID-19 or vaccine brands like Pfizer/BioNTech. Note that using keywords like “antibod” or “vaccin” allows to match all words including these sets of strings, such as, antibode, antibodes, vaccine, vaccines, vaccinations, and so on. We considered several different languages, such as, English, Russian, Chinese, and German. Afterwards, we manually inspected the listings to exclude false positives from the dataset, we categorized the listings in specific subcategories (e.g. specific brands), and we standardised the analysed attributes for the analysis. For example, we converted all prices to USD at the daily exchange rate at the time of observation.

**Table 1:** Search of COVID-19 vaccines. Keywords used to pre-select vaccine listings from the original dataset. Words are truncated to include different suffixes (e.g., vaccin yields vaccine, vaccination, vaccinate, etc.)

(i) Vaccine related set of keywords
antibod, vaccin, antidot, vacun, immun, Инокул, вакцин, прививк, Ревакцин, Инокул, 疫苗, 反, impfstoff, Gegenmittel
(ii) COVID-19 and brands related set of keywords
covid, corona, ковид, Коронавирус, Пандеми, Вирус, Спутник V, Инфекци, Симптом, 新冠病毒, 武汉肺炎, couronne, pfizer, astrazeneca, moderna

Such method is not feasible as more products are searched, because the number of listings to be manually annotated is too large. As already done in our previous work [10], we then limit our analysis to all listings mentioning COVID-19, using one of the following keywords: “corona virus”, “covid”, “coronavirus” either in the title or description. To analyse COVID-19 related products, we first pre-selected a subset of these listings mentioning keywords in specific categories, see Table 2, and then manually annotated these listings. With respect to our previous effort, we find a new product category, which we call *malware*, while no listing in the *ventilator* category was found. Then, we characterize all listings mentioning COVID-19 by means of Natural Language Processing techniques. We perform such analysis on the title, which contains essential information about the listing. First, we use doc2vec, a deep learning model that creates vector embeddings of sentences and paragraphs, to map the listing titles into high-dimensional numerical vectors. In particular, we make use of the specific *“paraphrase-mpnet-base-v2”* model implemented by the python package *sentence-transformers* [48], a pre-trained model which embeds sentences into a 768-dimensional vector space. In this space, semantically similar sentences are mapped in vectors close to each other, allowing for a quantitative way of detecting similar listings. In order to capture clusters of similar titles, we first need to reduce the dimension of the space without losing the information encoded in the distance between vectors. To this end, we use the UMAP algorithm [49] to map the 768-dimensional vectors to a 2D space, preserving its structure. We finally employ the *hDBSCAN* algorithm [50] (with minimum cluster size of 100 documents) to cluster these 2D vectors. We then label each cluster according to the category of products the listings refer to, manually inspecting the highest ranking words as ranked by the *tf-idf* algorithm, a statistical measure that evaluates how relevant a word is in a collection of documents. When not otherwise specified, we used default parameters.

While each listing had an associated url to determine its uniqueness, which allowed us to track listing over time, vendors receiving bad reviews sometimes put identical copies of the same listing online. To overcome this issue and correctly count the number of listings, we created a new identifier of unique listings. We considered two listings as unique if the same vendor was posting listings in the same market, having only small variations in the title. We also excluded listings with prices larger than 40, 000 USD. Vendors post listings at high price to hold sales of these relative items, with the expectation of offering it again in the future [51].

**Table 2:** COVID-19 products related keywords. Keywords used to pre-select listings selling COVID-19 related products before their manual annotation, organised by category.

Category	Keywords
Guides on scamming	guide, fraud, exploit, scam, loan, relief, scampage, cashout
Medicines	chloroquin, azithromycin, favipiravir, ritonavir, lopinavir, remdesivir, dexamethasone, ciprofloxacin, doxyciclin, oseltamivir, metronidazol, ivermectin
PPE	mask, glove, gown, surgical, sanitiser, sanitizer, ppe
Test	test kit, covid test, pcr test, antigen test, corona test, diagnostic, diagnosis
Web domain	https, www., http://, .com, .co.uk, .dk, .org, .info, .in, .net

## Results

### Vaccine listings

We start by analysing COVID-19 listings since November 2020. We found 248 unique listings offering vaccines and manually categorised them in three categories: *approved vaccines*, *unspecified vaccines* and *proofs of vaccination*. Listings in the *approved vaccines* category explicitly mentioned official vaccines, an example being the Pfizer/BioNTech vaccine that was offered at 500 USD on the Invictus marketplace, see Fig 5 in S1 File. Listings in the *unspecified vaccines* category instead referred to unbranded vaccines, for example by offering alleged unapproved vaccines well before official clinical trials were completed, as shown in Table 3 in S1 File. For instance, our previous analysis [10] found 34 listing advertising fake cures for COVID-19, including antidotes, vaccines, and allegedly curative recreational drug mixes. These listings were scam, since no official vaccine was approved in the considered time period. Listings in the *proofs of vaccination* category offered a fabricated certificate of COVID-19 vaccination, as the fake COVID-19 passport offered at 55 USD on the Hydra marketplace, see Fig 6 in S1 File with its English translation in Table 4 in S1 File. The *unspecified vaccines* category contained 94 listings, followed by the *proofs of vaccination* category with 80 and then the *approved vaccines* category with 74 listings. The *unspecified vaccines* category also has the highest number of vendors, with 61 offering these products across 13 different DWMs. Similar statistics for the other categories can be found in Table 9 in S1 File.

**Fig 1:**
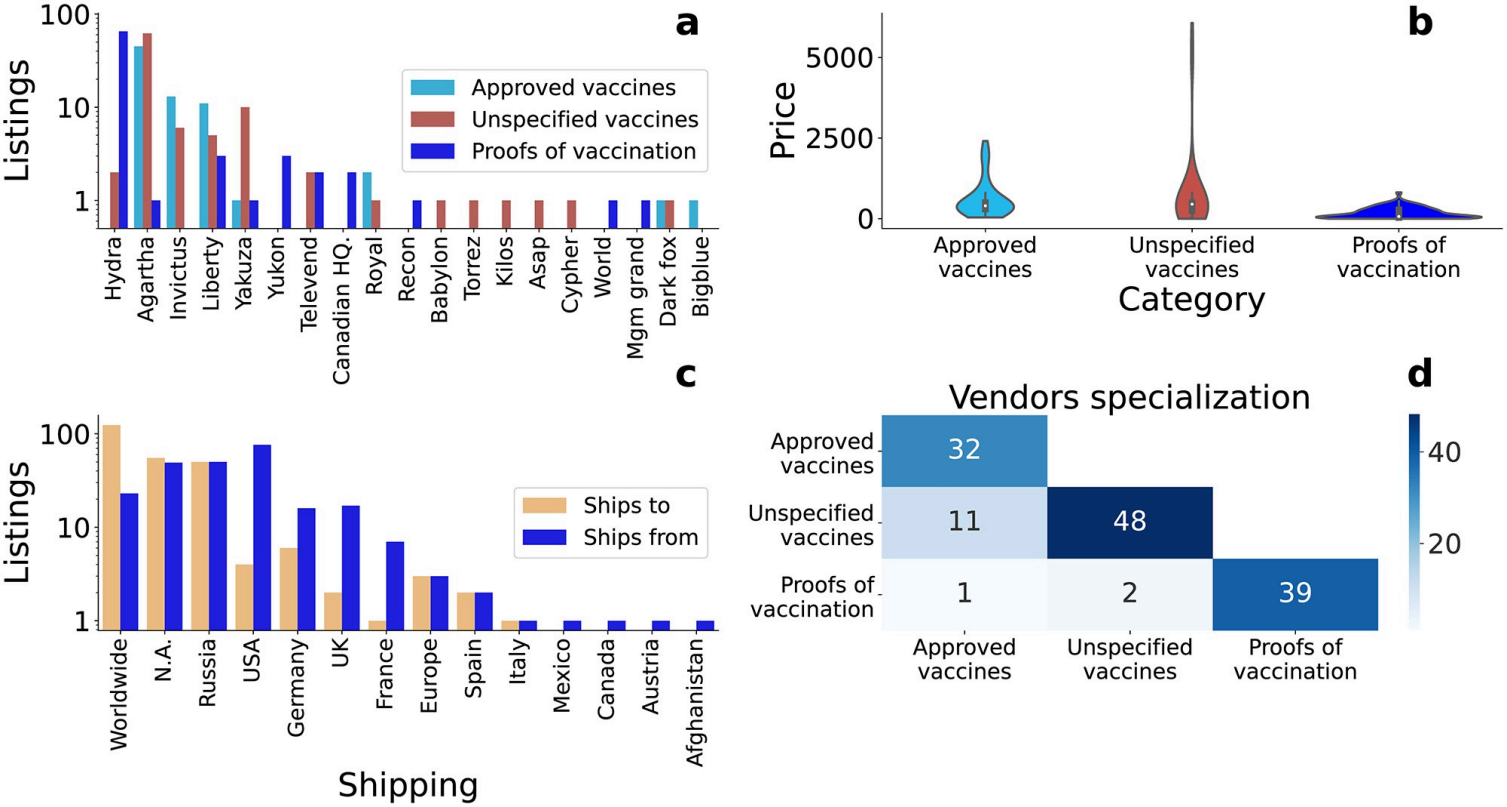
DWMs and COVID-19 vaccines. (a) Number of unique listings offered in each DWM. “BB. house” stands for Big brother house, while “Canadian HQ.” to The Canadian Headquarters. (b) Violin plot of the prices in USD at which vaccines were offered, showing the distribution of prices for the three categories. (c) Number of listings indicating where vaccines are declared to be shipped from and to. “N.A.” stands for not applicable and “Russia” for Russia and Eastern neighbouring countries. (d) Number of vendors offering a vaccine in a given category. Only the lower triangle of the matrix is shown because it is symmetric, where its diagonal represents vendors offering only listings in that category.

In Fig 1 we characterize the offer of these listings. We start by considering how the offer of vaccines was distributed across markets. The majority of vaccines were offered in the Agartha marketplace, with 108 listings, followed by Hydra with 67, which offered 65 out of the 80 fabricated COVID-19 vaccination certificates in our dataset. Fig 1(a) shows the category of listings offered by each DWM with at least one vaccine. 11 of these DWMs are specialized in offering only one category of listings, with one DWM only offering *approved vaccines*, 5 DWMs only offering *unspecified vaccines*, and 5 DWMs *proofs of vaccination*. Three DWMs, Agartha, Liberty, and Yakuza, offer at least one listing in each of the three categories considered. The DWMs specialization can be seen in Fig 9 in S1 File. Vaccine listings have a short lifetime on a DWM, with most listings that are offered for less than 25 consecutive days, see Fig 8 in S1 File. Such short lifetimes may be due to platform moderation, which in some cases explicitly prohibit such listings, supplies running out or even vendors taking down the listings because of bad reviews. However, such claims are not verifiable with our current dataset.

Regarding the price of vaccine listings, Fig 1(b) shows its distribution in the three categories under consideration. Listings in the *approved vaccines* category have prices ranging from 40 to 2,400 USD; listings in the *unspecified vaccines* category between 25 USD to 6,060 USD; and listings in *proofs of vaccination* category from less than 1 USD up to 814 USD. Proofs of vaccination were the cheapest products, probably because they consist of fake documentation (e.g., falsified COVID-19 passport). Price of *approved vaccines* listings varied depending on the vaccine brand offered, see Fig 11 in S1 File. The first listing in this category to be offered was the Pfizer/BioNTech vaccine at 1,000 USD. The other 44 listings offering the Pfizer/BioNTech vaccine proposed prices ranging from 200 to 2,400 USD. The Astrazeneca/Oxford vaccine, the second to be officially approved, was offered on DWMs since December 27, 2020. Only four listings offered this vaccine, ranging from 300 to 900 USD. The other approved vaccines offered on DWM were Moderna with 21 listings, Johnson&Johnson with four, Sputnik V with four, and Sinopharm with two. Their prices ranged from 40 to 2,000 USD. We speculate that one possible reason behind the skewness of these price distributions could be the presence of scam listings pretending to be selling these products at very low prices.

A natural next step is to analyse the geography of this trade, which we can do by looking at the shipping origin/destination information advertised in the listings. Most vendors declared that they would ship anywhere in the world, a behaviour that facilitates illicit trade. Vaccine warehouses were mostly in USA, followed by Germany and UK. Also, many listings do not declare any shipping information and all general shipping statistics are visible in Fig 1(c). In the 58% of the cases where no shipping information is declared, vendors invite potential customers to a direct interaction through Whatsapp, email, or phone. The percentage of listings where vendors suggest to initiate a direct interaction varies depending on the category considered. It happens for 78 (or the 84%) of listings in the *unspecified vaccine* category, for 64 (or the 85%) of listings in the *approved vaccine* category, and for only three listing in the *proofs of vaccination* category. This last low number is due to Hydra marketplace, which sells 64 proofs of vaccinations but whose vendors never shared their contact information.

Do these vendors sell multiple kinds of products related to vaccines? Or do they focus on a single category? Fig 1(d) shows that vendors offering *proofs of vaccination* were specialised, with only one vendor also offering *approved vaccines* and two unspecified vaccines. On the contrary, 11 vendors were offering both *vaccines* and *unspecified vaccines*. We did not observe any vendor offering listings in all three categories. Moreover, most vendors (tracked by username in the absence of PGP signatures) offer only one COVID-19 listing and trade in only one DWM, with the notable exception of a vendor, who had twelve listings in eleven different DWMs, as detailed in Fig 10 in S1 File.

**Fig 2:**
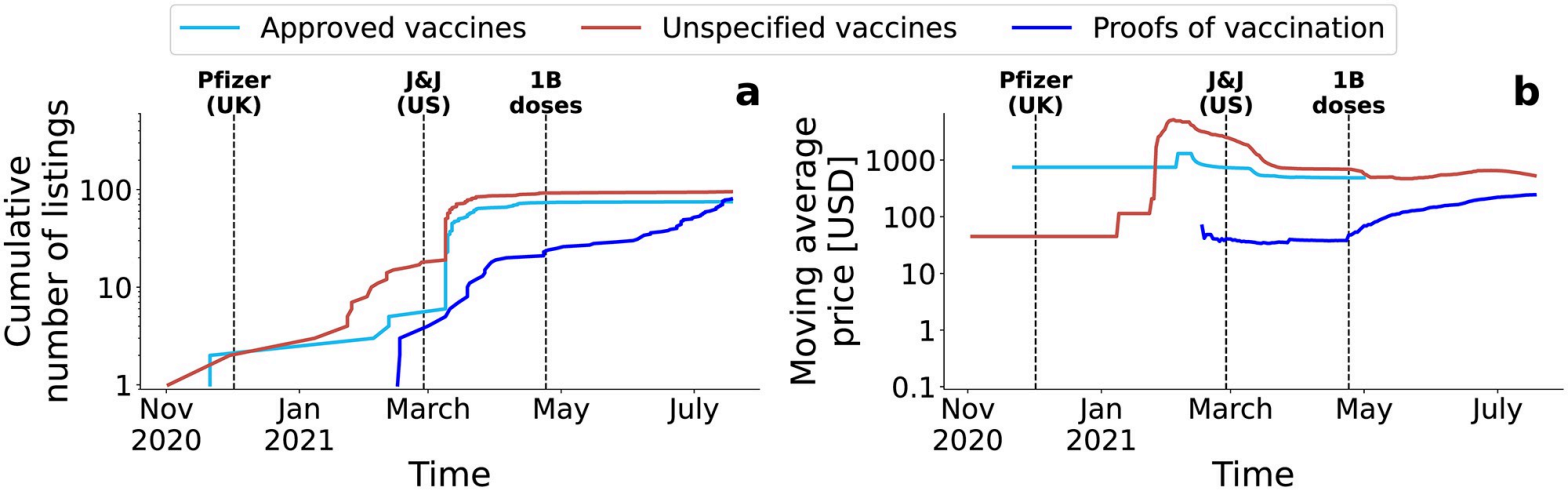
Temporal evolution of COVID-19 vaccine listings. (a) Cumulative number of listings over time in the three categories considered. (b) Average price over time in the same three categories, computed with a 90-days moving window. Vertical dashed black lines represent relevant pandemic events.

We now consider the time evolution of this offer of vaccine listings on DWMs, as shown in Fig 2. The evolution of vaccines on DWMs closely followed major COVID-19 related events, as shown in Fig 7 in S1 File and a sample of which is also shown on the background of Fig 2(a). Fig 2(a) shows that multiple vaccine listings were simultaneously present on DWMs when the first vaccination trials were undergoing, between March 16, 2020 and April 14, 2020 [52]. No more vaccine listings were present on DWMs from July 1, 2020, coincidentally with the end of the first wave of contagions in Europe (June, 2020 [53]). These listings reappeared on September 16, 2020, at the beginning of the second wave of infections that started in September 2020 [54]. Up to that moment, we detected only COVID-19 listings in the *unspecified vaccines* category. The first listing in the *approved vaccines* category was a Pfizer/BioNTech vaccine and was offered since November 17, 2020, two weeks before its first official approval on December 2, 2020 by the UK regulator MHRA [3]. A similar pattern was registered for the first AstraZeneca/Oxford vaccine listing on DWMs. It was offered on December 27, 2020, three days before the first official approval of this vaccine (by the UK) on December 30, 2020 [55]. The remaining approved vaccines, Johnson&Johnson, Moderna, Sputinik V, and Sinopharm, all appeared in the first half of March, when we started to monitor the Agartha marketplace. All *approved vaccines* listings disappeared on DWMs after May 1, 2021, albeit there may be other DWMs offering these products that are not part of our analysis. Since listings in the *unspecified vaccines* category continued to be observed until July 2021, we speculate that vendors were starting to have multiple vaccine brands, and they did not specify anymore which one are selling. For more details, see Fig 12(a) in S1 File. Listings in the *proofs of vaccination* category emerged on February 15, 2021, when airlines were encouraging governments to allow certificates of vaccinations to become a way to safely travel [56].

Finally, we looked at the temporal evolution of the average price of these listings. The three categories followed different trends, as visible in Fig 2(b). The price of *unspecified vaccines* was high between March and May 2020, when DWMs vendors likely tried to profit from the initial lack of COVID-19 medications [10]. Afterwards, their mean price has gradually decreased, meaning that the new listings appearing on DWMs were offered at progressively lower prices. However, the average price rose back to March levels in January 2021, when vaccinations campaigns around the world were starting. The availability of officially tested vaccines led to the emergence of listings advertising officially approved vaccines on DWMs since November 2020. The average price of these listings have floated over time between a few hundreds USD to more than a thousand. For more details, see Fig 12(b) in S1 File. Finally, the needs for a certificate of COVID-19 vaccination had mean-while increased, and so had the price of listings in the *proofs of vaccination* category. Vaccines certificates have gradually become mandatory in many countries, and especially for international travel, and their sale on DWMs confirms what researchers had hypothesised [57], warning against similar situations happening in the future.

### Other COVID-19 related products

**Fig 3:**
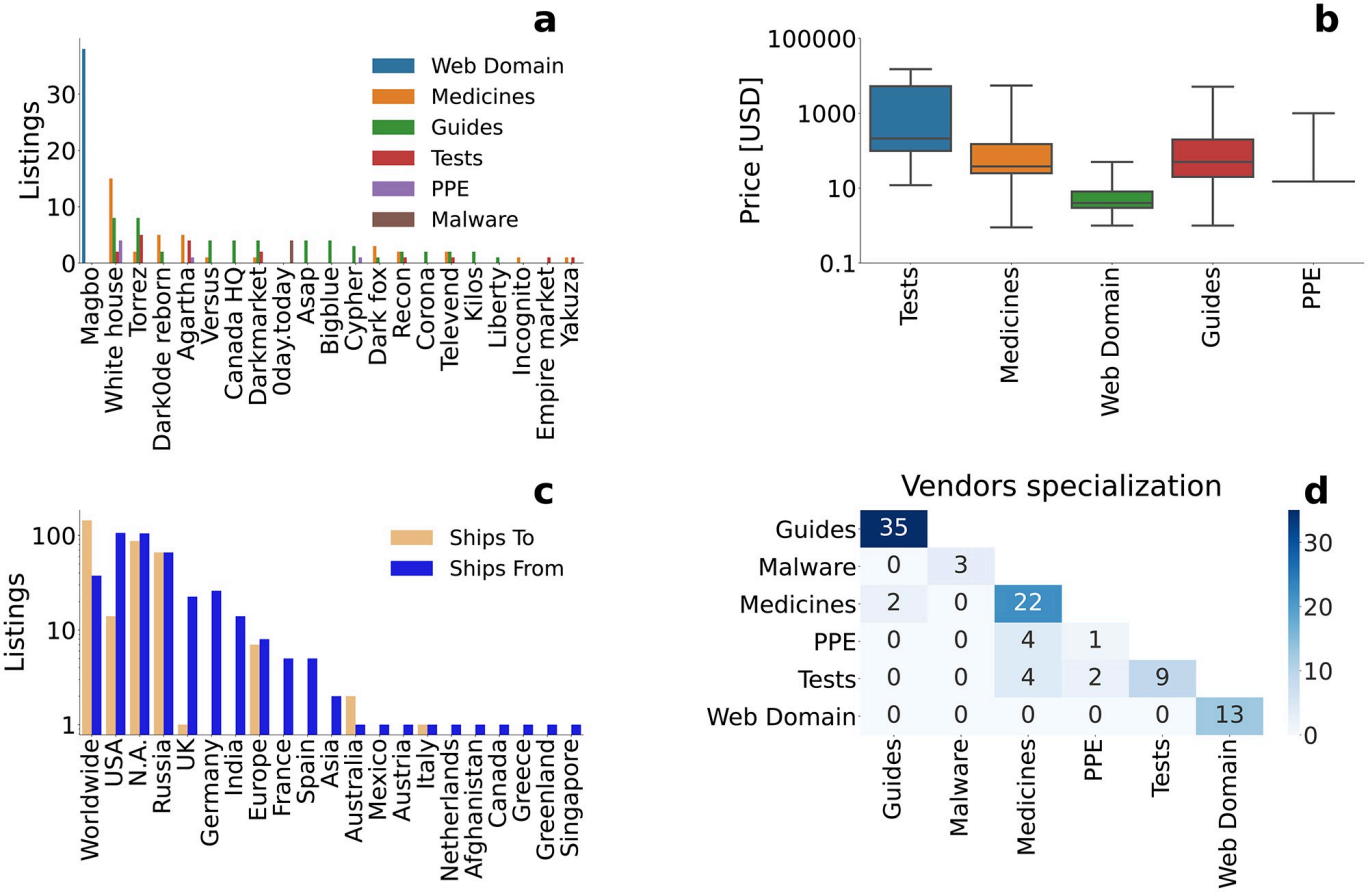
COVID-19 related products. (a) Break-down of COVID-19 related products by category and market. (b) Boxplot representing the price distribution of listings in each category. Horizontal lines represent the median value, box ends the first and third quartiles, and whiskers minimum and maximum values, respectively. (c) Number of listings indicating where COVID-19 related products are declared to be shipped from and to. “N.A.” stands for not applicable and “Russia” for Russia and Eastern neighbouring countries. (d) Number of vendors offering a COVID-19 related product in a given category. Only the lower triangle of the matrix is shown because it is symmetric, where its diagonal represents vendors offering only listings in that category.

DWMs have been a venue for the sale of other licit and illicit COVID-19 related products, like PPEs, tests, or medicines, as reported for the period from January to November 2020 [10]. Here, we monitor COVID-19 related products in the second part of the pandemic, between November 2020 and July 2021, see Table 10 in S1 File and Fig 3. Listings are divided in six different categories: *PPEs* represent healthcare objects like masks; *medicines* COVID-19 related medicines like hydroxychloroquine; *guides on scamming* are instructions on how to get relief funds; *tests* represent COVID-19 tests; *web domains* that are related to COVID-19 like “covidtest4you.com”; and *malware* represents malicious software to hack COVID-19 test or vaccination records software. Listings from these categories are offered in 21 DWMs, and are available in multiple markets. *Malware* and *web domains* are an exception because sold in two specific markets only. We find that *PPEs* and *medicines* have almost disappeared from DWMs w.r.t. previous observations [9, 10]. *PPEs* listings are mostly advertising bulk sales at high prices, coherently with the end of shortages of these products, while *medicines* listings, like hydroxychloroquine, are substituted by vaccines and present on DWMs in a lower number, with only 3 listings advertising Ivermectin [58]. On the contrary, *guides on scamming* were still present with comparable numbers, claiming to teach ways to access COVID-19 relief funds in different countries. Notably, the number of listings offering COVID-19 tests had also increased, with tests increasingly being required for travel or work. We also found 4 listings advertising malware to illicitly access official systems to record test results or even vaccinations.

Fig 3(a) shows the distribution of unique listings on each DWM offering them. These listings are concentrated in 4 DWMs, with the majority of them offering less than 5 listings. However, there is less category specialization w.r.t. what observed for the vaccines, with multiple markets offering listings in different categories. Prices are also very heterogeneous, with *test*’s median price highest at a few hundreds USD, and *web domain*’s lowest at just 4 USD, but also listings inside the same category ranging from 10 USD to 1000 USD in all categories but *web domain*, see Fig 3(b). In Fig 3(c), we show the origin and destination of the considered listings, as declared by vendors. The majority of listings declare to be shipping worldwide, while the United States is the country appearing the most as declared origin of the listings. Russia and Eastern neighbouring countries are both origin and destination, mainly because of *proof of vaccination* listings offered on Hydra, whereas UK, Germany and India appear almost only as countries of origin. Other countries/regions are declared, but less frequently. Fig 3(d) shows vendors specialization regarding COVID-19 related products. All categories show highly specialised vendors, except *PPE*, where only one vendor out of seven sells only in that category, and *tests*, where less than 55% of vendors sell only such products.

### Listings with COVID-19 mentions

In this section we extend our analysis to the offerings of products that mention Covid-19 in the title or body, without being directly related to pandemic-products, thus providing a richer assesment of the overall impact of COVID-19 on DWMs. We extend our previous analysis by considering listings appearing until July 2021 and by categorising the selected listings, providing a richer and deeper picture of how DWMs were indirectly affected by COVID-19.

**Fig 4:**
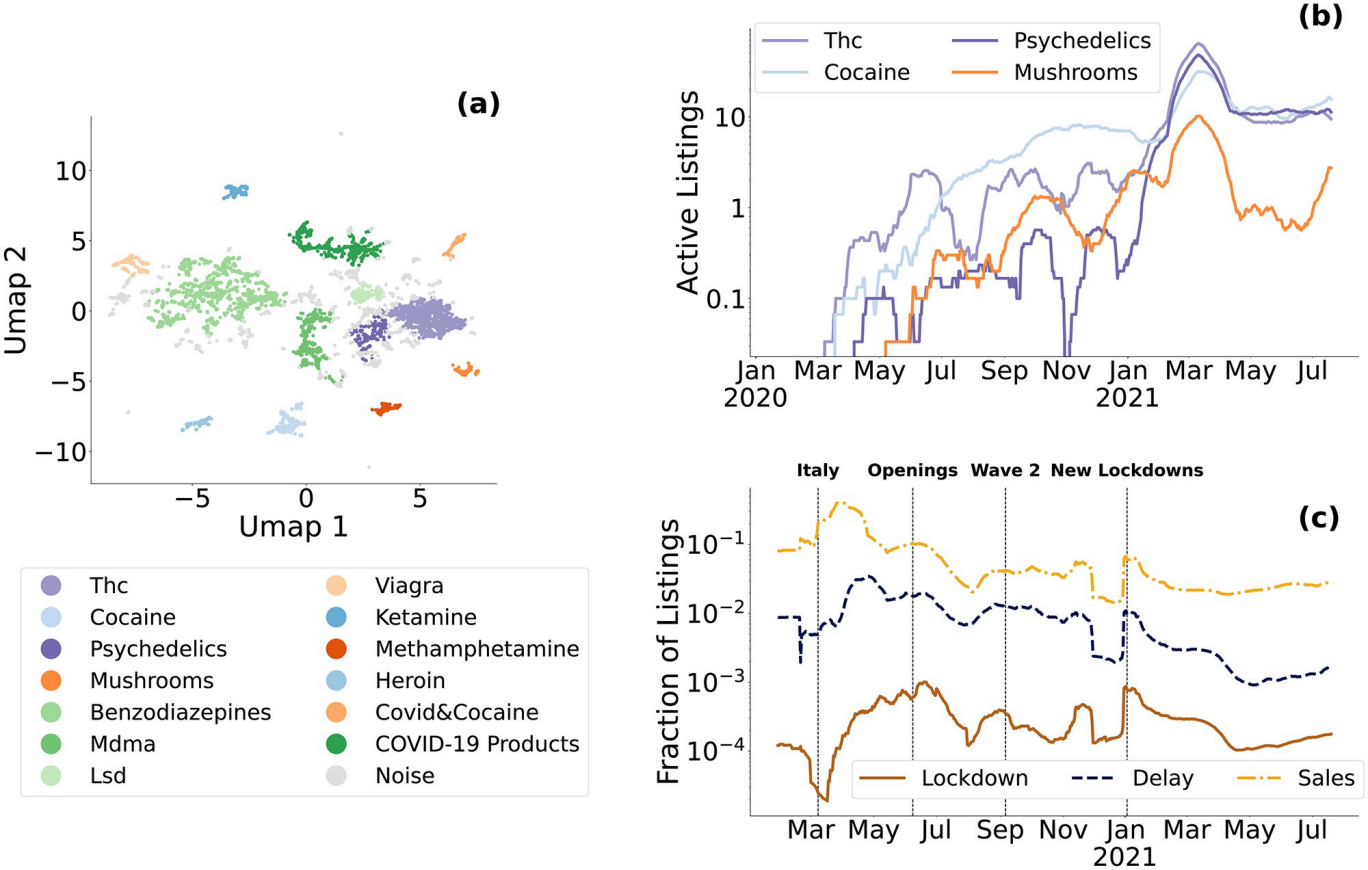
Characterization of COVID-19 mentions. (a) UMAP representation of doc2vec embeddings. hDBSCAN clustering finds 13 meaningful categories covering COVID-19 related products and all major drugs sold on DWMs. (b) 30-day rolling average of active listings in 4 categories of listings mentioning COVID-19: *thc*, *psychedelics*, *cocaine*, and *mushrooms*. (c) 30-day rolling average of fraction of previously identified drugs listings mentioning 3 different COVID-19 related themes: lockdowns, shipping delays and sales, with key pandemic events highlighted with vertical dashed black lines.

We characterise products mentioning COVID-19 with state-of-the-art Deep Learning based Natural Language Processing techniques [49, 50, 59], see Methods for more details. As shown in Fig 4(a), we find 13 different categories of listings corresponding to different kinds of products. In addition to the already discussed COVID-19 related products, only drugs appear to be mentioning COVID-19, while other traditional DWMs’ products like stolen IDs or credit card dumps don’t, showing which kind of goods reacted to, or where affected by, the pandemic. We then analyse the temporal evolution of these categories. We show the number of active listings for 4 large categories in Fig 4(b), while all other categories are shown in Fig 13 in S1 File. Drugs show an overall increasing trend throughout the whole period. Different categories, however, display different fluctuations in time, showing how different goods behave in an heterogeneous way with respect to COVID-19. For example, at the end of our covered period we can see *thc* and *psychedelichs* showing a flat trend, while *cocaine* and *mushrooms* are increasing.

While it is not possible to understand the reasons behind each single temporal trend, we can gain more insights on why drugs are increasingly mentioning COVID-19 by investigating which themes are recurrent in these listings. In Fig 4(c), we count mentions over time of three different set of keywords, which can be used as general proxies of the indirect impact of the pandemic. We considered (i) *lockdown*, by tracking listings mentioning “lockdown” or “quarantine,”; (ii) *delay*, by monitoring listings using keywords “delay” or “shipping problem,”; and (iii) *sales*, by searching for “sale,” “discount,” or “special offer.” For instance, sellers may mention lockdown to justify the lack of international deliveries or problems with their supply. Similarly, sellers may mention possible delays due to COVID-19 related restrictions and supply issues, or promote sale during the economically-challenging COVID-19 period [10, 60]. *Lockdown* mentions are always lower than the other two themes, peaking in summer 2020 but staying always lower than 20%. *Delay* mentions instead rapidly increase during the first months of the pandemic, and have been oscillating around 60% of the listings since then, showing how drug vendors have been warranting possible delays throughout the whole observed period, confirming what’s already been independently shown for the first phase of the pandemic [60]. Finally, *sale* mentions show larger fluctuations between as low as 15% to even 80%. In particular, we can observe peaks related to the pandemic at the beginning of key COVID-19 related events: lockdowns in March/April 2020, in Summer 2020 coincidentally with openings in the western world, in October 2020 when the second wave started hitting Europe, and in February 2021 when the Delta variant started spreading in the world. By looking at mentions of these themes across all listings in our dataset, we find that overall mentions of *lockdown*, *sales*, and *delay*, have decreased since the beginning of the pandemic, validating our finding that drugs-related listings are the product most impacted by COVID-19, see Fig 14 in S1 File.

Automatic keyword search has allowed us to uncover macroscopic trends, but it fails to capture finer details which can only be uncovered by in-depth looks at the texts of the listings. We therefore resort to a qualitative analysis of their descriptions. First, we already noticed that mention of delays in drug listings are still frequent, amounting to 56% of the listings. While vendors generally preemptively mention possible delays due to COVID-19, we find numerous mentions of USA based vendors blaming USPS for these, as shown in one example reported in Table 5 in S1 File: “THE USPS IS UNDERFUNDED AND MAY BECOME UNRELIABLE COMPARED TO THE PAST! (ESPECIALLY DURING COVID-19 AND HOLIDAYS!)”. These claims reflect widely reported issues with the United States Postal Services since June 2020 [61]. Moving away from delays, we find that 10% of vendors mention COVID-19 by ensuring potential clients that they are taking all necessary safety measures when preparing the deliveries. An example of this is reported in Table 6 in S1 File. Finally, we find listings mentioning limited stocks due to the pandemic, as shown in Table 7 in S1 File, where the vendor claims that“stocks are almost exhausted by Corona Covid 19”.

## Discussions and conclusion

In this paper we have studied how DWMs have responded to the ongoing COVID-19 pandemic along multiple dimensions, with a special focus on the sale of COVID-19 vaccines. Covid-19 vaccines have indeed been a key element of the exit from the emergency phase of the pandemic, and regulators and international agencies warned of their possible illicit trade on DWMs and the associated health risks [7, 8]. The covered period, ending at the end of July 2021, included the second phase of the pandemic, i.e., when vaccines became available. We have identified a sharp increase in the number of listings selling vaccines and proofs of vaccinations, from 34 between January and November 2020 (when no vaccine had been released yet) to 248 after, including officially approved vaccines like Pfizer or Moderna. Vaccine related listings have replaced other previously observed COVID-19 related products (e.g. PPE and hydroxychloroquine), whose presence has been steadily decreasing since November 2020. While assessing the overall COVID-19 impact through the analysis of listings explicitly mentioning COVID-19, we have found that drugs were the most affected traditional DWMs product. Our results extended previous analyses [9, 10] on the impact of COVID-19 on DWMs both in terms of duration of the monitored period and of breadth of the analysed products.

A key contribution of the present work is the study of the interplay between DWMs and the COVID-19 pandemic, after the official approval of vaccines. It was previously shown that, when a product is in shortage in the regular economy, or public attention is focused on it, listings advertising its sale appear on DWMs. For instance, this is what happened for PPE and hydroxychloroquine during the first phase of the pandemic. Since in the observed period these products were easily available on regulated markets, we coherently detect that these products disappeared in the second phase of the pandemic. In late 2020, we have seen the same pattern with vaccines, which appeared around the time of their official approval, reflecting the claims of other mass media news [62–64]. They then spiked at the beginning of 2021, to be later replaced by fabricated proofs of vaccinations with the increasing need of vaccine passports and green passes [65, 66]. Mentions of lockdowns, delays, and sales followed similar dynamics, with spikes observed in the first phase of the pandemic in 2020 and their mentions steadily decreasing during the second phase. However, we found that drugs listings mentioning COVID-19 increased in time, with numerous mentions of delays and sales, some of which are related to stock shortages and increase in health security measures, as unveiled by our qualitative analysis. Our results confirm what was already observed for other external shocks creating extraordinary demand for specific goods. For instance, it has been shown that the restriction of access to hydrocodone combination products, the most commonly prescribed opioid, in the United States in 2014 caused DWMs to step in to meet the unaddressed demand [67, 68].

A limitation of the present work is that, while the number of DWMs simultaneously monitored over time is greater than most previous studies, we cannot ensure that all DWMs were surveyed. In fact, the number of active DWMs is constantly changing due to closures or new openings [16] and obtaining full coverage is challenging due to the active efforts of DWMs to obstruct research studies and law enforcement investigations. Moreover, our study is limited to what vendors advertise on these platforms, as we have no data on actual purchases to quantify how many people have been endangered by this phenomenon. Future work, relying on backend servers seized during police takedowns of DWMs, could improve on our study by overcoming these limitations.

The diffusion of illicit vaccines on DWMs, together with the simultaneous decrease of PPEs and medicines, confirms the link between product shortages, public attention and listings on DWMs. This phenomenon has the potential to pose a serious public health threat, as DWMs have become increasingly easier to access, resilient to police closures [16] and shown to be a catalyst for decentralized peer to peer trading between buyers and sellers of illicit items [34]. The purchase of unregulated, and possibly fraudulent, health related items on DWMs poses a concrete health risk for the buyers. Moreover, the availability of fake vaccination or testing records risks to undermine public health measures implemented by numerous countries worldwide, and calls for more investigation of this phenomenon for the current and future pandemics. By highlighting how vaccines appear on DWMs, our analysis may help raise awareness of the phenomenon and support the effort of law enforcement agencies to contain it by repeating past successful approaches [27–33]. Furthermore, our results call for more investigation of DWMs to anticipate such dangers in future public health crisis.

## Supporting information

S1 File (PDF)
