# Supplementary material for: Vaccines and more: The response of Dark Web marketplaces to the ongoing COVID-19 pandemic
Source: PLoS One. 2022 Nov 10;17(11):e0275288. doi: 10.1371/journal.pone.0275288 (PMC9648775; doi:10.1371/journal.pone.0275288)
Supplement: S1 File — (PDF) [file pone.0275288.s001.pdf]

Examples of detected listings

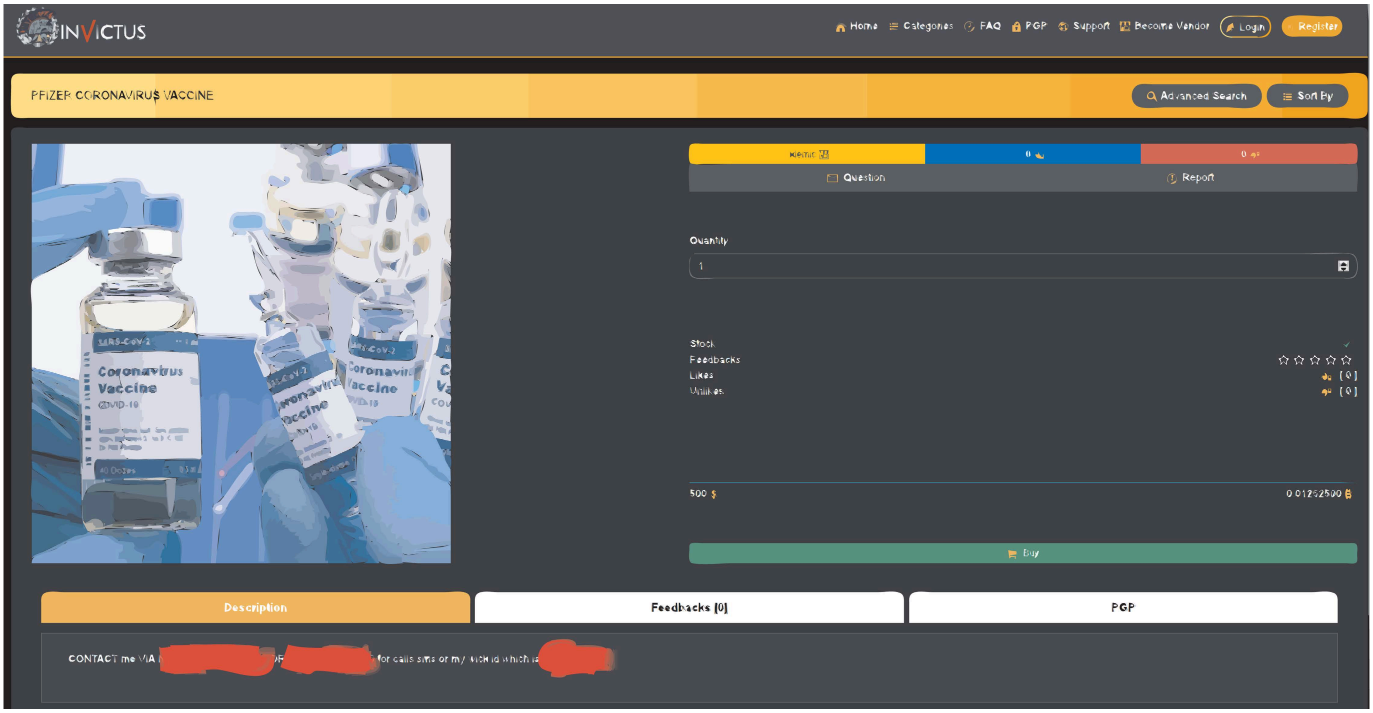

Figure 5. Pfizer/BioNTech vaccine offered on Invictus. Screenshots of a listing in the *approved vaccines* category offering the Pfizer/BioNTech vaccine at $500 on the Invictus marketplace. We removed the contact information of the vendor, who invites the potential customer to have a direct contact. The screenshot was taken on February 6, 2021.

Table 3. Generic vaccine offered on a DWM. Example of a listing in the *unspecified vaccines* category offering a generic vaccine, which does not specify the producer. Personal information of the vendor are hidden with # symbols.

Title	COVID-19 antidote from china. offering at 15k USD
Body	the covid-19 current massacre is supposed to have ended by now. while the who is trying to be selfish with human life, we are trying to save the lives. the real virus is the leaders. this vaccine should be used just once on one person and basically the giveaway price i put here is nothing compare your life. get your vaccine now in time. you can buy from me and resell at your price. contact me for more details. email: ##### wickr: ##### telegram: ##### kik: #####
Price	15,000 USD
Shipping from/to	N.A.
Vendor	#####
DWM	DarkBay

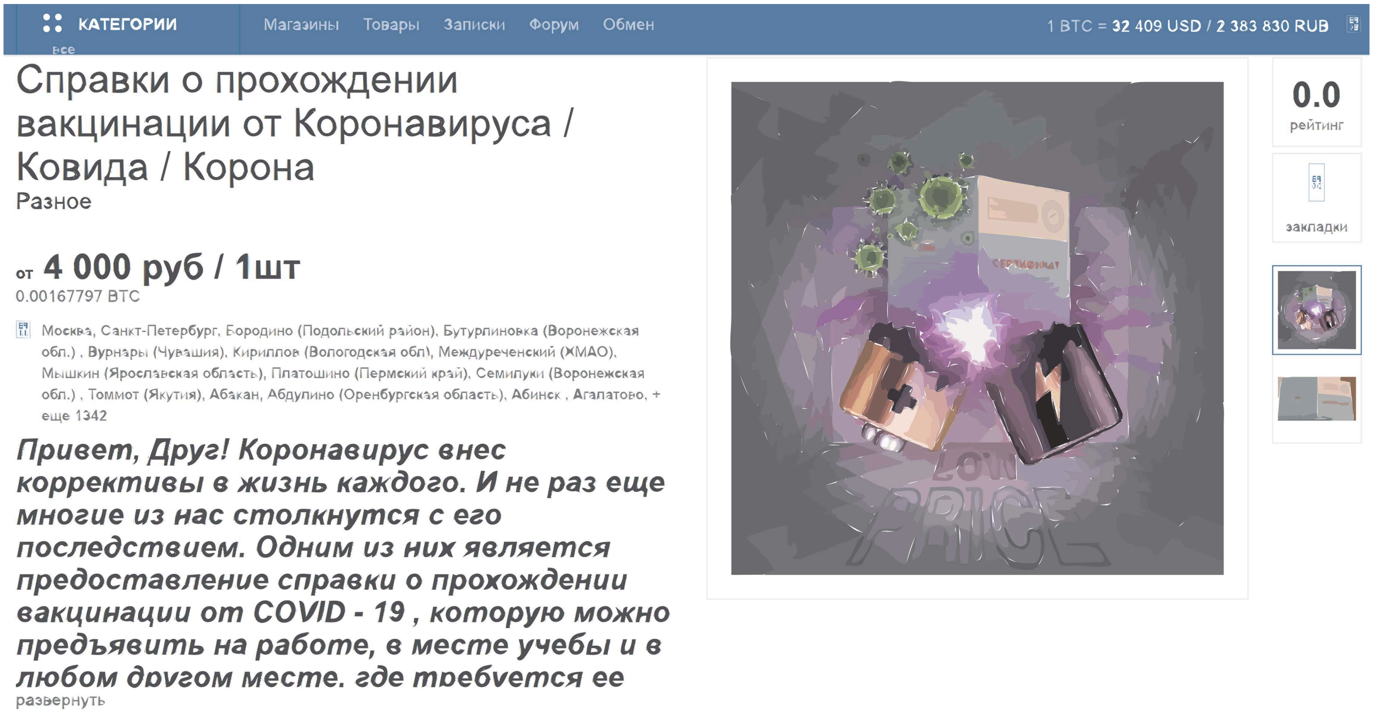

Figure 6. Proof of vaccination offered on Hydra. Proof of vaccination offered at 4,000 rubles (55 USD) detected on the Hydra marketplace. The original language of this listing is Russian and its translation in the English language is given in Table 4. The screenshots were taken on February 6, 2021.

Table 4. Translation of the proof of vaccination offered on a Hydra. English translation of the listing in Fig 6(b). We use Google Translator to translate the text from Russian to English.

Title	Coronavirus / Covid / Corona vaccination certificates
Body	Hello Friend! The coronavirus has made adjustments to everyone's life. And more than once many of us will face its consequences. One of them is the provision of a COVID-19 vaccination certificate, which can be presented at work, at the place of study and in any other place where it is required. To order, you need to indicate your full name, date of birth, date of the issued document. If you need a non-Moscow institution, you will need to pay extra for the production of the necessary seals. Production time is 2-7 days. Delivery when sent by courier will have to be paid for upon receipt. It is possible to send by registered or regular mail, then we will take on this heavy burden. An ordinary letter has no track, therefore, until we receive the letter, we will remain in the dark about its fate.
Price	55 USD
Shipping from/to	Russia and neighbouring Eastern countries/Russia and neighbouring Eastern countries
Vendor	#####
DWM	Hydra

Table 5. Example of drug listing mentioning COVID-19, and problems with the United States Postal Services (USPS).

Title	(10) 30mg adderall pressed pills: us - us
Body	Adderall is used in the treatment of attention deficit hyperactivity disorder (ADHD) and narcolepsy. It is also used as an athletic performance enhancer, cognitive enhancer, appetite suppressant, and recreationally as an aphrodisiac and euphoriant. It is a central nervous system (CNS) stimulant of the Phenethylamine class.<br>You MUST Be A Minimum Of 18 Years of Age<br>For Research Purposes Only Not For Human Consumption Refund Policy ALL SALES ARE FINAL!<br>REFUNDS WILL NO LONGER BE ISSUED DUE TO SCAMMING! RESHIPS ARE ALWAYS AVAILABLE ON A CASE BY CASE BASIS, AND USUALLY ONLY WHEN A TRACKING NUMBER NEVER ORIGINALLY SCANS, OR GETS STUCK FOR 15+ DAYS.<br>NO reships will be sent in the event of a tracking status of RETURN TO SENDER or UNDELIVERABLE AS ADDRESSED.<br>Reships DO NOT qualify if a package status is marked as Delivered or indicates the package is In Transit to its destination. If the package is in the system, please wait it out for the package to arrive. THE USPS IS UNDERFUNDED AND MAY BECOME UNRELIABLE COMPARED TO THE PAST! (ESPECIALLY DURING COVID-19 AND HOLIDAYS!)<br>Use a real name and address for your package. If a package is stuck IN TRANSIT for a few days, and a tracking number is given to you, please call USPS to locate it. THOUGH I MAY CARRY NON SCHEDULED RESEARCH CHEMICALS, DO NOT CLAIM TO KNOW THE CONTENTS. NO PACKAGES WILL EVER REQUIRE A SIGNATURE!
Price	59.13 USD
Shipping from/to	USA/USA
Vendor	#####
DWM	Dark0de Reborn

Table 6. Example of drug listing mentioning COVID-19, and ensuring safety measures are taken.

Title	black diamond og sfv og shake popcorn thc
Body	thank you so much for shopping with us we are confident you'll love your order while your here take a moment to browse through our vendor page to see the many great strains bulk orders and emeraldgallipot promotional offers we have to offer<br>what we offer fast communication all msg are answered within hrs fast delivery product will be shipped on the next business day after order confirmation via usps priority mail stealth packaging vacuum sealed odorless sterile packaging package tracking available upon request three days after order confirmation full refund replacement if tracking confirms package seized lost<br>what we ask please provide your full address immediately in pgp format in buyer's note use your full name and double check your address deliveries that tracking confirms lost because of errors in provided information are not available for refund or replacement<br>all shipping addresses must be in the following format name john doe address nameless ln city state zip city xx<br>please finalize asap upon receiving package please leave a positive rating if you are unhappy with your order please tell us we are happy to work with you to satisfy your needs<br>strain highlights black diamond og indica dominant hybrid backberry kush diamond og thc<br>flavor aroma a cross between blackberry and diamond og its flowers have a glittery trichome covering and purple coloring that make it a beautiful gem to look at the strains aroma is musky and earthy almost like a deep red wine euphoric effects black diamond is known to cause fits of giggles and is a great strain for hanging out with friends and creative expression<br>medical benefits ideal for patients who need strong medication but still want to be active and sociable this strain tends to make consumers extremely hungry making it a good choice for those looking to increase their appetite just make sure you have some snacks on hand<br>san fernando valley og sativa og kush direct thc<br>flavor aroma sfv og by cali connection is a sativa dominant hybrid that is as the name indicates this og kush relative originates from californias san fernando valley although their names are barely distinguishable sfv og kush is actually the afghani crossed child to sfv og leading with aromatic notes of earthy pine and lemon<br>euphoric effects creates a long lasting head haze and full body effect that leaves you feeling happy and relaxed without damping your energy<br>medical benefits great for patients who need strong pain relief but dont want to be stuck on the couch<br>note we here at the emeraldgallipot take our customers safety as our highest priority and to help protect you against the spread of the coronavirus all packages we send are being thoroughly sterilized with a mild disinfectant and bleach solution prior to shipping for your protection stay safe out there
Price	50 USD
Shipping from/to	USA/USA
Vendor	#####
DWM	Torrez

Table 7. Example of drug listing mentioning COVID-19, and “stocks are almost exhausted by Corona”.

Title	grams speed paste normal quality
Body	When you place an order you agree with our conditions!<br>Offer: 5 Grams Speed Paste Normal Quality<br>This product is made from high grade washed A-Oil<br>Purity: 45% up to 55%<br>Approximately 20% of the weight is lost during the drying process<br>For any questions feel free to contact us, we are happy to help you!<br><br>Welcome to #### The best speed (amphetamine) products on the market! We sell from the normal quality till the highest quality you can get! Our sending fits every mailbox! We ship from Monday till Friday!<br>We ship from Germany and we know how to ship! It is important for us that all orders arrive in all safety!<br>SHIPPING TIME Europe: 2 to 7 Business days Worldwide: 4 to 20 Business days<br>REFUND and RESHIP If orders not arrive please send us a message and we find a solution. In case of non-arrival, we will reship 50% or a 50% refund. Mistakes made in the address-format we will never reship or refund. New buyers without any order history we never refund or reship.<br>Please give us some great feedback if you are happy with us!<br>AmphetamineCowboys<br><br>UP-DATE 13-02-2021<br>Dear customers, From today 13-02-2021 we will go into vacation mode for 10 days until 23-02-2021. We do this because we have a lot of money in escrow and our stocks are almost exhausted by Corona Covid 19. New stocks are on the way but unfortunately it is slowing down due to Covid bullshit. We do not want to disappoint. We will receive new stocks next week so that we can continue on 23-02-2021. Of course all accepted orders have been shipped including today! We are online every day for all your questions about the shipped orders or for any other questions. Hoping for some understanding from you, we will be back soon on 23-02-2021. All be safe and hope to see you soon!<br>Sehr geehrte Kunden, Ab heute 13.02.2021 werden wir fr 10 Tage bis zum 23.02.2021 in den Urlaubsmodus wechseln. Wir tun dies, weil wir viel Geld im Treuhandkonto haben und unsere Aktien von Corona Covid 19 fast erschpft sind. Neue Aktien sind auf dem Weg, aber leider verlangsamt sie sich aufgrund von Covid-Bullshit. Wir wollen nicht enttuschen. Wir werden nchste Woche neue Aktien erhalten, damit wir am 23.02.2021 weitermachen knnen. Natrlich wurden alle angenommenen Bestellungen auch heute noch versendet! Wir sind jeden Tag online fr alle Ihre Fragen zu den versendeten Bestellungen oder fr andere Fragen. In der Hoffnung auf ein Verstndnis von Ihnen werden wir bald am 23.02.2021 zurck sein. Alle sind in Sicherheit und hoffen, Sie bald zu sehen!<br>Chers clients, partir d’aujourd’hui 13/02/2021, nous passerons en mode vacances pendant 10 jours jusqu’au 23/02/2021. Nous faisons cela parce que nous avons beaucoup d’argent en squestre et que nos actions sont presque puises par Corona Covid 19. De nouvelles actions sont en route mais malheureusement, elles ralentissent cause des conneries de Covid. Nous ne voulons pas dcevoir. Nous recevrons de nouveaux stocks la semaine prochaine afin de pouvoir continuer le 23/02/2021. Bien sr, toutes les commandes acceptes ont t expdies, y compris aujourd’hui! Nous sommes en ligne tous les jours pour toutes vos questions sur les commandes expdies ou pour toutes autres questions. En esprant une comprhension de votre part, nous serons de retour bientt le 23/02/2021. Soyez tous en scurit et esprons vous voir bientt!
Price	17 USD
Shipping from/to	Germany/Worldwide
Vendor	#####
DWM	White House

DWMs offering COVID-19 vaccines

Table 8. List of DWMs analysed.

Type of products	DWM
COVID-19 vaccines	Agartha, Asap, Babylon, Bigblue, Cypher, Dark fox, Hydra, Invictus, Kilos, Liberty, Mgm grand, Recon, Royal, Televend, The Canadian Headquarters, Torrez, World market, Yakuza, Yukon
COVID-19 related products	0day.today, Agartha, Asap, Bigblue, Corona, Cypher, Dark fox, Dark0de reborn, Darkmarket, Incognito, Kilos, Liberty, Magbo, Recon, Televend, The canadian headquarters, Torrez, Versus, White house, Yakuza
COVID-19 mentions	0day.today, 24HoursPPC, ASAP, Agartha, Amigos, Apollon Marketplace, Asean, Atshop, Auction DB, Aurora, Babylon, Big Brother House, BigBlue, Blackhole, CannaHome, Cannabay, Cannazon, Cartel, Cindicator, Connect, Corona, Cypher, Dark Fox, Dark Leak Market, Dark0de Reborn, DarkBay/DBay, DarkMarket, Database, Deep Sea, Deepsy, DutchDrugz, Empire Market, Exchange, FSpros, Faceless, Flugsvamp 3.0, Fullzbuy, Genesis marketplace, HeinekenExpress, Hexablaze, Hookshop, Hydra, Incognito, Invictus, Kilos, Liberty, MEGA Darknet Market, MGM Grand, MagBO, Market Deepmix, Metropolis, Monopoly, Mouse In Box, Namaste LSD, Olux, Opiate Connect, Pentagon, Plati.market, Psylab Seeds, RNJLogs, Recon Search Engine, Royal, Russian Market, SEOclerks, Scans24, Sellix, Shoppy.gg, Silk Road 3.1, Silk Road 4, Tea Horse Road, Televend, The Canadian HeadQuarters, Tor Market, Torrez, UAS, Versus, WTN Market, White House, Willhaben, World Market, Xleet, Yakuza, Yellow Brick marketplace, Yukon

Table 9. Vaccine listings detected on DWMs. Some vendors and DWMs offer vaccines that belong in more than one category.

Category	Unique istings	Vendors	DWMs
Unspecified vaccines	94	61	13
Approved vaccines	74	44	7
Proofs of vaccination	80	42	10
Total	248	134	19

Supplementary information

Figure 7. Summary of key events related to DWMs and COVID-19. Availability of listings offering vaccines on dark web marketplaces (top), together with main COVID-19 related events of the vaccination campaign (bottom).

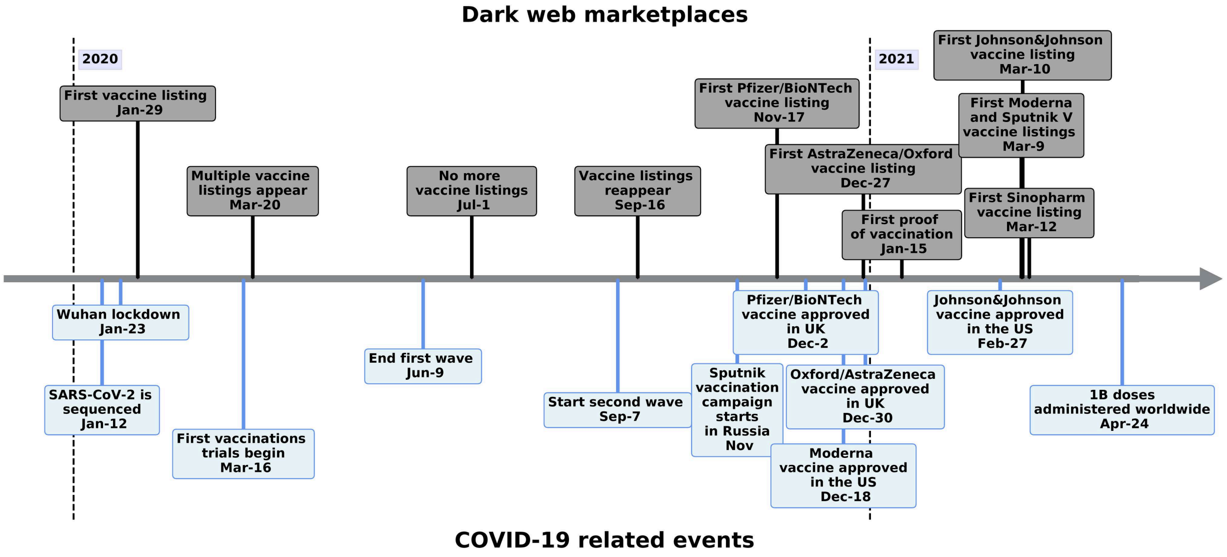

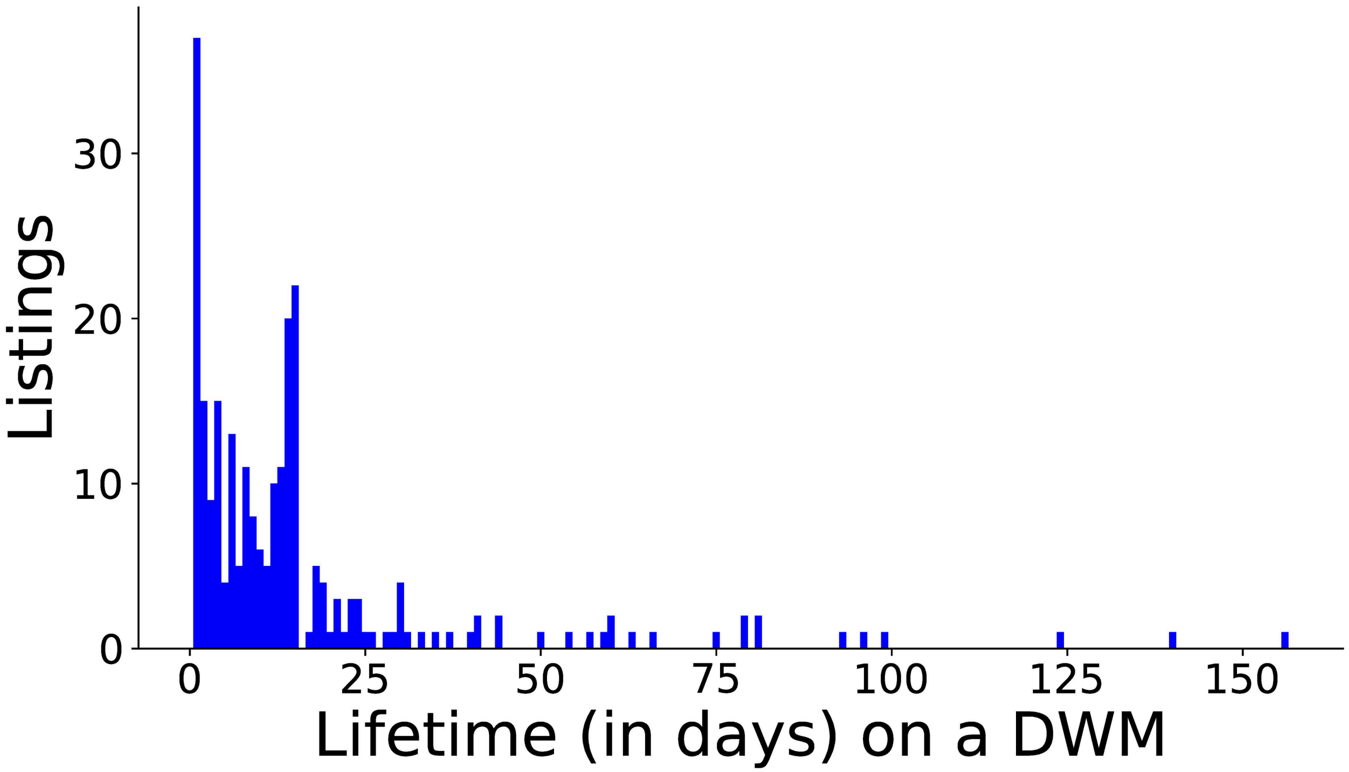

Figure 8. Lifetime of listings on a DWM. Number of days during which listings were active on a DWM.

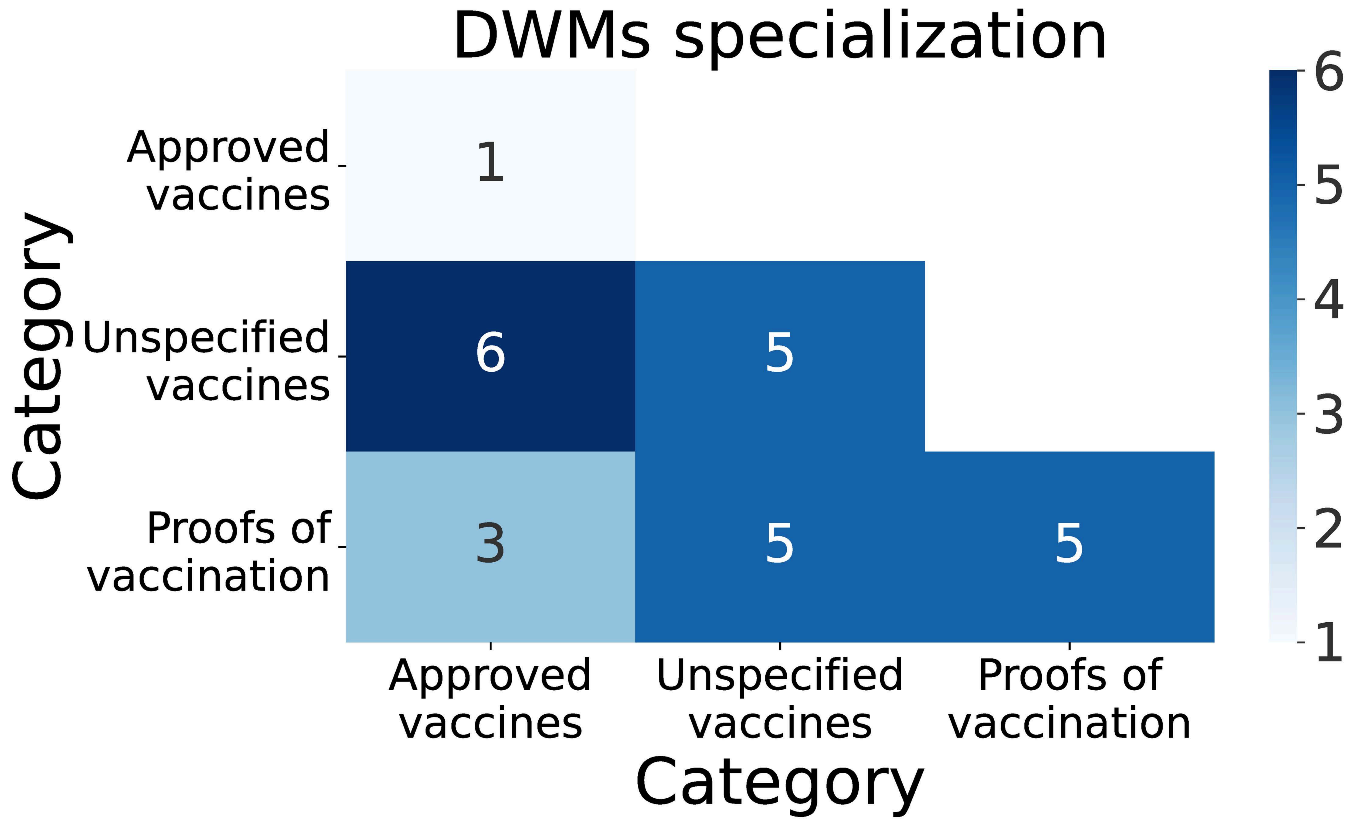

Figure 9. Categories of vaccines offered on DWMs. Number of DWMs offering a vaccine in a given category. Only the lower triangle of the matrix is shown because it is symmetric, where its diagonal represents vendors offering only listings in that category.

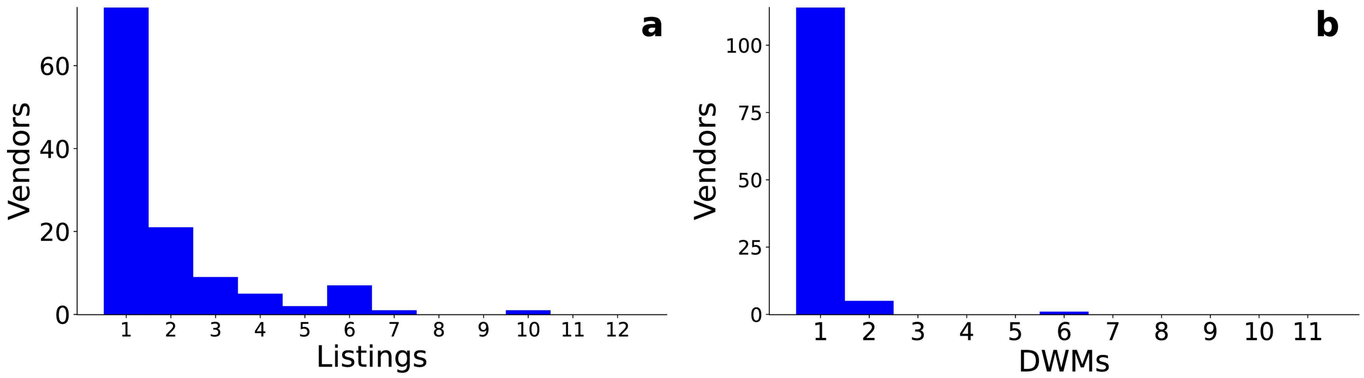

Figure 10. Vendor statistics. Histograms representing the number of vendors offering a certain amount of vaccines listings, in panel (a), and the number of vendors trading in a given amount of DWMs, in panel (b).

Table 10. COVID-19 related products offered on DWMs. Availability of COVID-19 related products since November 2020.

Category	Unique listings	Observations	Median price [USD]	Vendors	DWMs
Guides on scamming	50	885	50	36	15
Malware	4	19	NaN	3	1
Medicines	40	367	38.00	27	13
PPE	6	36	15.00	3	3
Test	17	85	211.12	11	8
Web domain	38	184	4.00	13	1

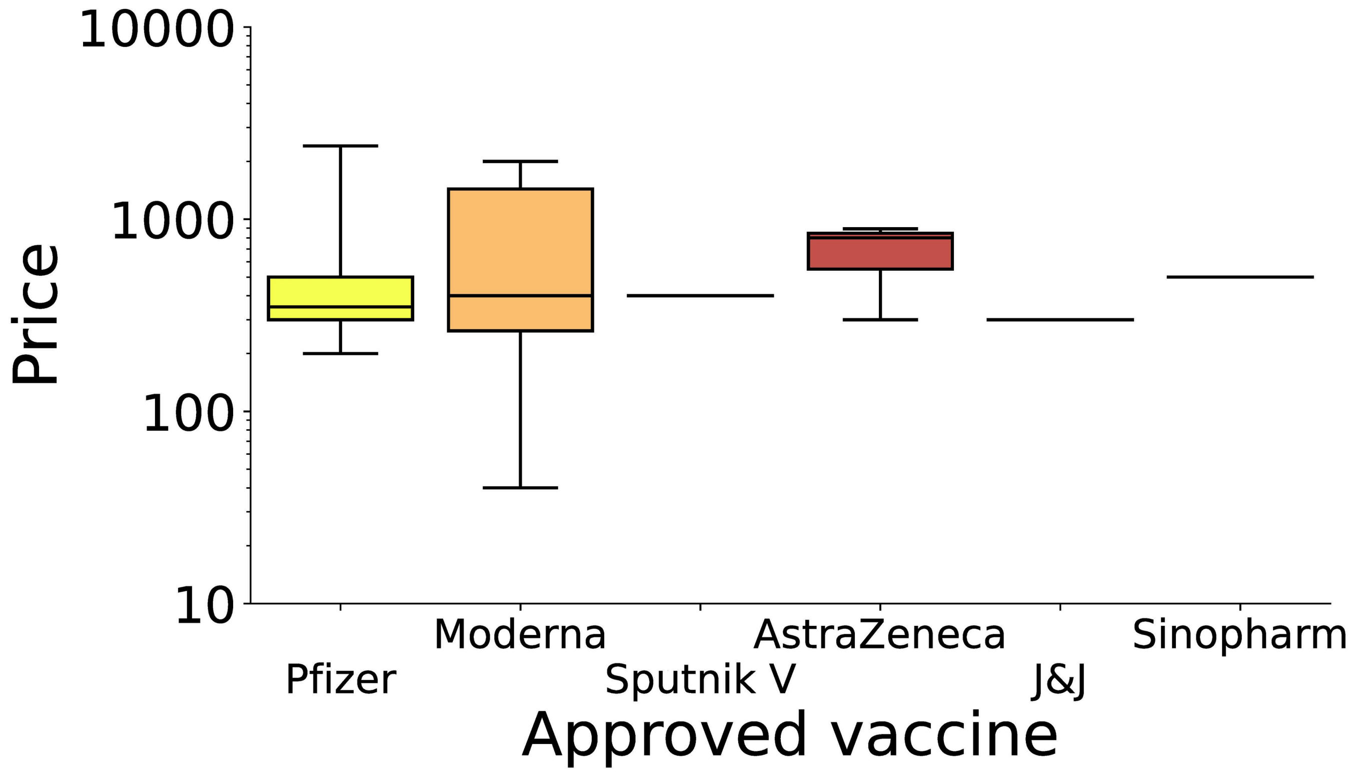

Figure 11. Price of COVID-19 *approved vaccines.* Boxplots of the prices in USD at which vaccines were offered. (a) Price of listings in the three categories considered. (b) Focus on the listings offering approved vaccines. "J&J" stands for Johnson&Johnson. Horizontal lines represent the median value, box ends the first and third quartiles, and whiskers minimum and maximum values, respectively.

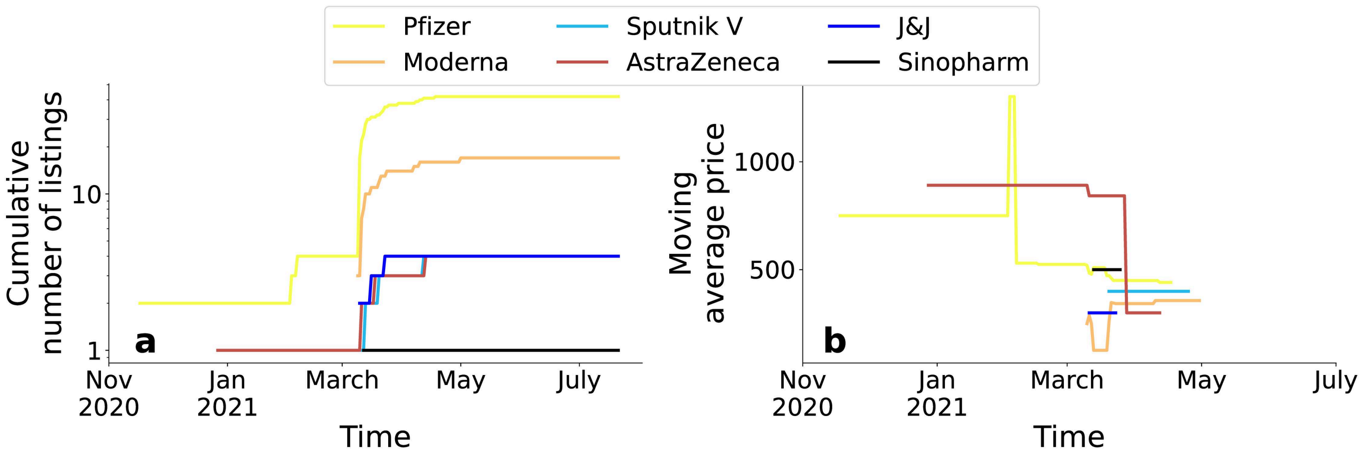

Figure 12. Temporal evolution COVID-19 *approved vaccines.* (a) Cumulative number of listings over time. (b) Average price over time, computed with a 90-days moving window."J&J" stands for Johnson&Johnson.

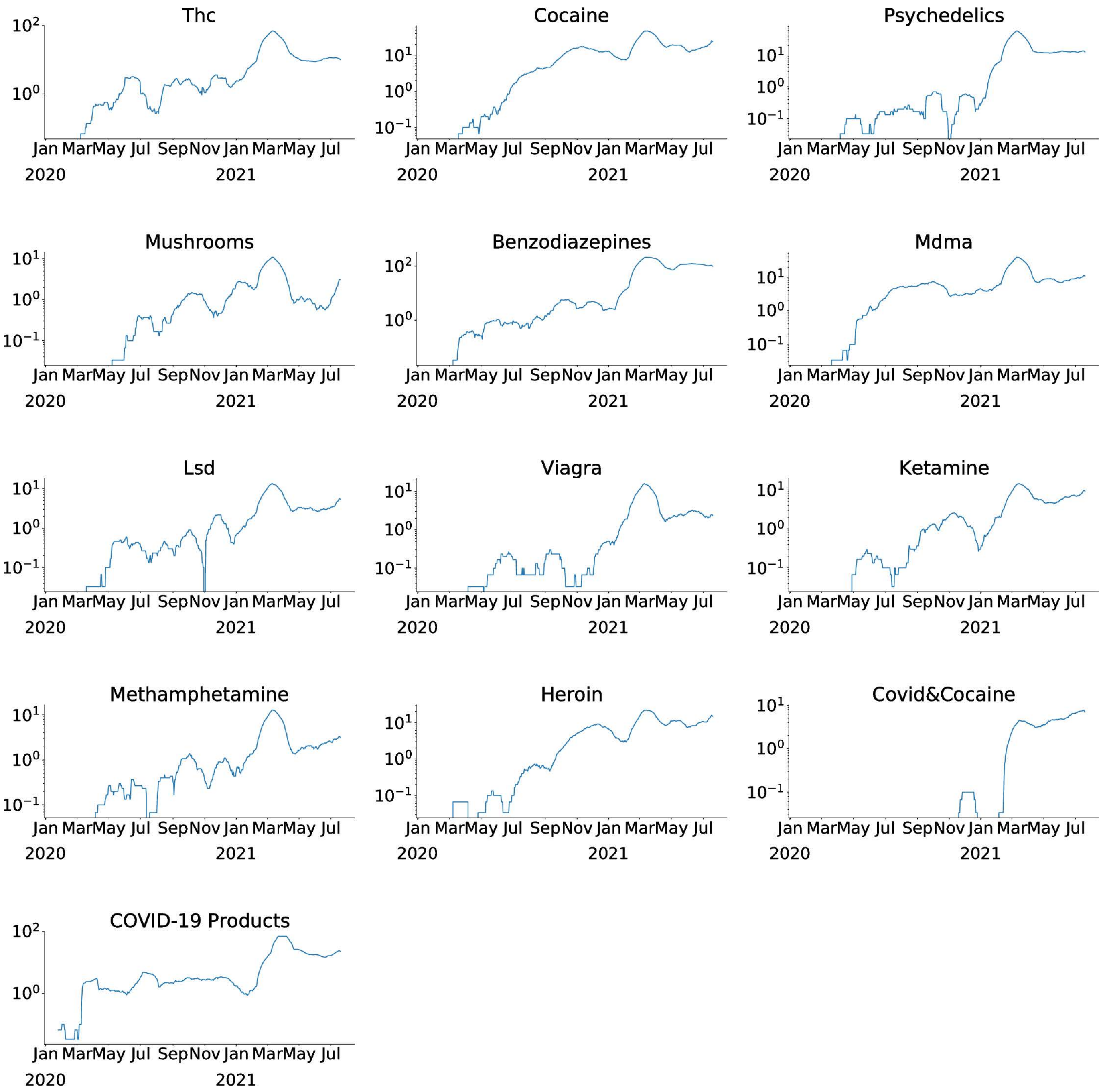

Figure 13. Time evolution of products mentioning COVID-19. Number of active listings in time in each product category, according to the clustering described in the main text.

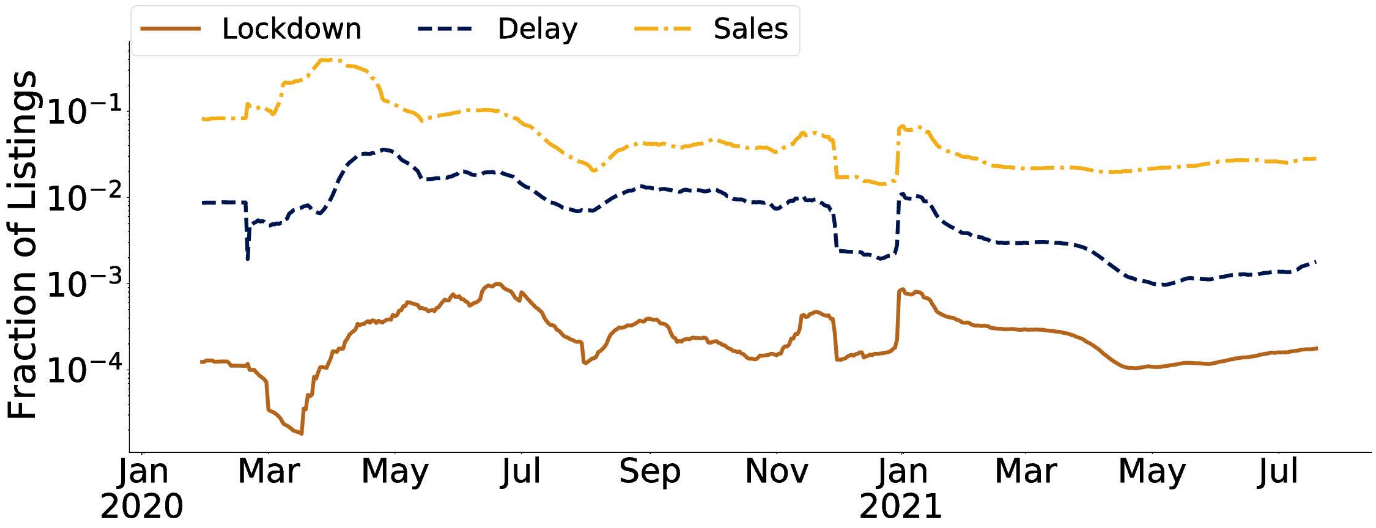

Figure 14. Time evolution of fraction of all listings mentioning COVID-19 related themes.
